# Enhanced Biofilm Formation by ICU-Associated *Stenotrophomonas maltophilia* Isolates: A Potential Contributor to Persistence and Clonal Dissemination

**DOI:** 10.3390/microorganisms14071471

**Published:** 2026-07-03

**Authors:** Giovanni Di Bonaventura, Giovanni Gherardi, Martina Barchitta, Antonella Agodi, Arianna Pompilio

**Affiliations:** 1 Department of Medical, Oral, and Biotechnological Sciences, “G. D’Annunzio” University of Chieti-Pescara, 66100 Chieti, Italy; arianna.pompilio@unich.it; 2 Center of Advanced Studies and Technology, “G. D’Annunzio” University of Chieti-Pescara, 66100 Chieti, Italy; 3 Antibiotic Resistance and Special Pathogens Unit, Department of Infectious Diseases, Istituto Superiore di Sanità, 00119 Rome, Italy; giovanni.gherardi@iss.it; 4 Department of Medical and Surgical Sciences and Advanced Technologies “G.F. Ingrassia”, University of Catania, 95123 Catania, Italy; martina.barchitta@unict.it (M.B.); antonella.agodi@unict.it (A.A.)

**Keywords:** *Stenotrophomonas maltophilia*, biofilm formation, intensive care unit, cystic fibrosis, cross-transmission, molecular epidemiology

## Abstract

*Stenotrophomonas maltophilia* is an emerging multidrug-resistant opportunistic pathogen in intensive care units (ICUs) and cystic fibrosis (CF), where biofilm formation may favor persistence, device-associated colonization/infection, and clonal dissemination. This study compared biofilm formation, clonal relatedness, biofilm phenotypes, and motility in 37 ICU-associated and 42 CF-associated *S. maltophilia* isolates. Biofilm formation on polystyrene was quantified by crystal violet assay and expressed both as absolute biomass and as a growth-normalized Biofilm Index, calculated to account for differences in planktonic growth. Genetic diversity was assessed by pulsed-field gel electrophoresis, while swimming and twitching motility were evaluated using agar-based assays. ICU isolates showed a higher prevalence of biofilm formation, greater biofilm biomass, and higher growth-normalized Biofilm Index values than CF isolates. They also displayed lower genetic diversity and more frequent cross-transmission, supporting the circulation of selected hospital-associated lineages. Conversely, CF isolates showed greater heterogeneity and a more complex biofilm pattern, consistent with adaptation to a distinct chronic airway environment. Motility was not associated with biofilm formation, suggesting that the enhanced biofilm phenotype of ICU isolates is not explained by swimming or twitching alone. Overall, these findings support a setting-specific model in which enhanced biofilm-forming capacity may contribute to *S. maltophilia* ICU persistence and clonal dissemination, highlighting the need for targeted surveillance and careful device management.

## 1. Introduction

*Stenotrophomonas maltophilia* is an opportunistic Gram-negative pathogen increasingly recognized as a relevant cause of healthcare-associated infections, particularly among critically ill and immunocompromised patients [1]. Although it has traditionally been regarded as a microorganism of relatively low intrinsic virulence, its clinical importance has progressively increased because of its ability to colonize vulnerable hosts, persist in hospital environments, and cause difficult-to-treat infections [2]. In intensive care units (ICUs), *S. maltophilia* has been reported among the most frequently isolated microorganisms from ICU-acquired pneumonia in both Italy and Europe [3]. The clinical interpretation of its isolation, especially from respiratory samples, remains challenging because colonization and infection may overlap in critically ill patients exposed to mechanical ventilation, invasive devices, prolonged hospitalization, and broad-spectrum antimicrobial therapy [4,5].

*S. maltophilia* is also considered an emerging pathogen in patients with cystic fibrosis (CF), where impaired mucociliary clearance, chronic airway inflammation, repeated antibiotic exposure, and polymicrobial communities may favor persistence [6]. Repeated or long-term antipseudomonal therapy may create ecological conditions that facilitate the recovery of intrinsically resistant non-fermenting Gram-negative bacteria, including *S. maltophilia* [7]. Chronic isolation of *S. maltophilia* from CF airways has been associated with lower lung function in some cohorts, although its overall clinical impact remains debated and may depend on host factors, disease severity, co-infecting microorganisms, and duration of colonization [8]. Thus, ICU and CF represent two distinct clinical and ecological settings in which *S. maltophilia* may persist under different selective pressures.

A major feature contributing to the clinical relevance of *S. maltophilia* is its intrinsic multidrug resistance. This species shows reduced susceptibility to several antimicrobial classes through multiple mechanisms, including low outer-membrane permeability, chromosomally encoded β-lactamases, multidrug efflux systems, aminoglycoside-modifying enzymes, and adaptive responses to antimicrobial stress [9]. These determinants of resistance restrict therapeutic options and may contribute to persistence during antimicrobial exposure [10]. In addition to conventional planktonic resistance, *S. maltophilia* can form biofilms, a lifestyle that further increases tolerance to antimicrobials and host defenses [11]. Biofilm-associated cells are embedded within an extracellular matrix and may exhibit altered metabolism, reduced penetration by antimicrobials, stress-adapted phenotypes, and an increased ability to survive unfavorable environmental conditions [12].

Biofilm formation is particularly relevant to microorganisms that can attach to living and non-living surfaces, including epithelial tissues, plastic materials, and medical devices [11]. We previously showed that *S. maltophilia* can grow as antibiotic-resistant biofilms [13]. In CF isolates, biofilm formation appears to be a highly conserved phenotype, with reported frequencies ranging from 76.9% to 100% depending on strain collection and experimental conditions [14]. This phenotype has been observed not only on abiotic surfaces, such as polystyrene, but also on CF-derived epithelial monolayers, suggesting a possible role in airway persistence and adaptation [15,16]. In this context, biofilm formation may contribute to long-term colonization of the chronically inflamed CF airway, although biofilm biomass measured in vitro cannot fully reproduce the complexity of the CF lung environment [17].

The ability of *S. maltophilia* to form biofilm is multifactorial and involves bacterial surface properties, extracellular structures, and environmental conditions. Fimbriae and other adhesin-like structures have been implicated in attachment to both epithelial cells and abiotic surfaces [18]. Cell-surface hydrophobicity and motility have also been associated with adherence and biofilm formation on polystyrene, supporting the use of abiotic-surface models to explore strain-dependent differences in biofilm-forming capacity [19]. In addition, flagella, type IV pili, lipopolysaccharide-related components, extracellular polymeric substances, outer-membrane structures, and regulatory systems involved in stress adaptation and cell–cell communication may contribute to different phases of biofilm development [18]. Swimming motility may facilitate access to surfaces and initial colonization, whereas twitching motility may contribute to surface-associated movement and biofilm architecture [19]. However, the relationship between motility and biofilm biomass is not necessarily linear, because biofilm development depends on multiple strain-specific and environmental determinants.

In the ICU setting, biofilm formation may have additional epidemiological relevance. *S. maltophilia* is well adapted to humid environments and has been implicated in water-related hospital outbreaks [20]. Devices that supply drinking water to ICU patients have been suggested as potential reservoirs for biofilm-associated *S. maltophilia* [20]. Faucets and other water-associated hospital niches have also been linked to transmission events in neonatal intensive care and hematology units [21,22]. These observations support the hypothesis that biofilm formation on abiotic surfaces may facilitate environmental persistence and indirect transmission in healthcare settings. Nevertheless, direct evidence linking biofilm-forming clinical isolates to environmental reservoirs requires dedicated surveillance studies and cannot be inferred from clinical isolates alone.

Molecular epidemiology can help clarify whether biofilm-forming isolates belong to heterogeneous populations or to successful clonal lineages. In hospital settings, the recovery of genetically related isolates from different patients may suggest cross-transmission or exposure to a common source, particularly when supported by temporal and spatial links [23]. In contrast, a highly diverse population may reflect repeated independent acquisition, within-host diversification, or adaptation to patient-specific ecological conditions [24]. Therefore, combining biofilm phenotyping with molecular typing may provide useful insights into the potential relationships among biofilm formation, persistence, and clonal dissemination.

Despite the increasing interest in *S. maltophilia* biofilm biology, the biofilm-forming ability of ICU-associated clinical isolates remains insufficiently characterized. Limited information is available on whether ICU isolates differ from CF isolates in biofilm biomass, distribution of biofilm phenotypes, and clonal structure. The present study was therefore aimed at investigating the in vitro ability of ICU-associated *S. maltophilia* strains to form biofilms and comparing this ability with that of strains isolated from CF patients. To this end, biofilm formation, clonal relatedness assessed by pulsed-field gel electrophoresis, and the distribution of biofilm phenotypes among epidemic and sporadic isolates were analyzed in both patient populations. Because motility may contribute to early surface colonization and biofilm development, swimming and twitching motility were also evaluated in ICU-associated isolates.

## 2. Materials and Methods

### 2.1. Bacterial Strains and Growth Conditions

Clinical and microbiological features of *S. maltophilia* clinical strains enrolled in this study are listed in Table 1. A total of 79 strains were collected over one year. Forty-two strains were isolated from respiratory specimens of CF patients admitted to Bambino Gesù Hospital of Rome. According to the CDC guidelines [23], the strains were classified as “probable pathogens” because the patient had symptoms and signs of infection at the site of isolation, but the culture yielded polymicrobial growth.

Thirty-seven *S. maltophilia* strains were isolated from several samples (blood, bronchial aspirate, urinary/venous catheters, pharyngeal and nasal swabs, swabs from surgical superficial incision) of patients admitted to the ICU of five ICUs in Catania, Italy. Considered patterns of *S. maltophilia* ICU-acquisition were as follows: (i) carriage on admission, (ii) colonization of sterile sites, and (iii) infections during ICU stay [pneumonia, bloodstream infections (BSIs), central venous catheter (CVC)-related BSIs, surgical site infections (SSIs), and urinary tract infections (UTIs)] [1,24,25].

All strains were identified as *S. maltophilia* by conventional biochemical tests (API 20-NE System; BioMérieux, Marcy-l’Étoile, France) or by the Phoenix system (Becton, Dickinson and Company, Pont de Claix, France). Stock cultures were maintained at −80 °C in a Microbank preservation system (Pro-Lab Diagnostics, Biolife Italiana S.r.l., Milan, Italy) until use. As needed, stocks were thawed and subcultured twice on Mueller-Hinton Agar (MHA) (Oxoid S.p.A.; Garbagnate M.se, Milan, Italy) for 24 h at 37 °C to assess purity and restore the original phenotype.

A standardized inoculum was prepared to perform all assays. Briefly, an overnight culture (37 °C, 16 h) was prepared in Trypticase Soy Broth (TSB) (Oxoid S.p.A) and adjusted for optical density measured at 550 nm (OD_550_) corresponding to approximately 1 × 10^7^ CFU/mL.

### 2.2. Biofilm Formation Assay

Two hundred microliters of the standardized inoculum were dispensed into independent wells of a sterile 96-well flat-bottom polystyrene tissue culture plate (Iwaki; Bibby Scientific Italia, Riozzo di Cerro al Lambro, Milan, Italy). Bacteria were incubated at 37 °C for 24 h in a closed, humidified plastic container. The spent TSB was then discarded, and non-adherent bacteria were removed by washing the wells three times with sterile phosphate-buffered saline (PBS; pH 7.3) (Sigma-Aldrich; Milan, Italy). Quantification of biofilm formed on polystyrene was assessed by a spectrophotometric method, as previously described [26], with minor modifications. After removing the PBS washing solution, biofilm samples were fixed by incubation for 1 h at 60 °C, then stained for 5 min at RT with a 0.1% Hucker crystal violet solution (Sigma-Aldrich). The wells were then rinsed with distilled water to remove the excess stain and dried at 37 °C for 30 min. Biofilms were destained by treatment with 200 μL of 33% glacial acetic acid (Sigma-Aldrich) for 15 min, and the optical density was measured at 492 nm (OD_492_) using an Infinite 200 Pro spectrophotometer (Tecan Group Ltd., Männedorf, Switzerland). The low cut-off for biofilm formation was chosen as 3 standard deviations (SDs) above the mean of control wells not seeded with bacteria [26]. A strain was classified as: “non-producer” (OD_492_ < 0.200), “weak-producer” (0.200 ≤ OD_492_ < 0.400), “moderate-producer” (0.400 ≤ OD_492_ < 0.800), or “strong-producer” (OD_492_ ≥ 0.800) [27].

To account for potential differences in planktonic growth among the tested strains, a Biofilm Index was calculated as OD_492_ biofilm biomass divided by OD_600_ measured after 24 h of incubation, before washing. Biofilm-producer categories were assigned using the established OD_492_-based cut-offs, whereas the Biofilm Index was used as a complementary analysis to control for growth-related bias.

### 2.3. Motility Assays

Swimming and twitching motility assays were performed as previously described [28], with modifications. (i) Swimming assay. A single colony was inoculated onto swimming agar (10 g/L tryptone, 5 g/L NaCl, 3 g/L agar; Oxoid S.p.A.). After incubation at 37 °C for 24 h, swimming motility was measured as the diameter of the growth zone. (ii) Twitching assay. A single colony was inoculated into the medium (1% TSB + 1% agar; Oxoid S.p.A.) until it reached the bottom of a Petri dish. After 72 h of incubation at 37 °C, the agar layer was removed, and the bacterial growth zone attached to the plastic surface was stained with crystal violet. Twitching motility was expressed as the diameter of the stained zone.

### 2.4. Macrorestriction Analysis and Genetic Relatedness

Genetic diversity among *S. maltophilia* isolates from CF and ICU patients was assessed by pulsed-field gel electrophoresis (PFGE) as previously described [1,29,30,31], with minor modifications. Bacterial cells were embedded in PIV buffer (10 mM Tris [pH 8.0], 1 M NaCl; Sigma-Aldrich). Plugs were incubated with lysozyme (1 mg/mL; Sigma-Aldrich) overnight at 37 °C and then with proteinase K (0.1 mg/mL; Sigma-Aldrich) overnight at 50 °C. DNA was digested with *XbaI* (25 U/mL; Sigma-Aldrich) for 20 h at 35 °C. Electrophoresis was performed in 0.5× TBE buffer (Sigma-Aldrich) under the following conditions: switching time, 5–35 s; temperature, 12 °C; run time, 20 h; voltage, 6.0 V/cm; included angle, 120°. Gels were stained with ethidium bromide and visualized under UV illumination.

Isolates with indistinguishable PFGE patterns were assigned to the same PFGE type and subtype. Those differing by 1 to 3 bands were considered genetically related and assigned to the same PFGE type, whereas isolates differing by 4 or more bands were considered genetically unrelated and assigned to different PFGE types. Genetic distance matrices were used to generate dendrograms by the unweighted pair-group method with arithmetic mean (UPGMA) using NTSYS-PC version 1.8 (Exeter Software, East Setauket, NY, USA) [8]. The diversity index was calculated as the number of distinguishable genotypes in the population divided by the population size [32]. Cross-transmission was assumed when indistinguishable isolates were recovered from patients treated in the ICU during overlapping periods or within 7 days [33,34].

For biofilm phenotype comparisons, isolates were classified as “epidemic” when they belonged to a PFGE type shared by two or more isolates from different patients. Isolates showing a unique PFGE profile within the study population were classified as possibly “sporadic”. Thus, “epidemic” indicated clonal clustering within the study collection, while “sporadic” indicated genetically unrelated single-isolate PFGE profiles.

### 2.5. Statistical Analysis

Each experiment was carried out in triplicate and repeated in three independent experiments. Continuous variables were compared using an unpaired *t*-test for two-group comparisons and a one-way ANOVA or a Kruskal–Wallis test for comparisons involving more than two groups, depending on the data distribution. Proportions were compared using Fisher’s exact test. Associations between biofilm variables and continuous clinical parameters were assessed by Pearson correlation/simple linear regression. Statistical analyses were performed using GraphPad Prism version 9.00 (GraphPad Software Inc., San Diego, CA, USA), and *p*-values < 0.05 were considered statistically significant.

## 3. Results

### 3.1. Patterns of S. maltophilia ICU Acquisition

Thirty-seven out of 79 *S. maltophilia* strains tested were from 28 ICU patients. Patterns of *S. maltophilia* ICU acquisition were described in Table 1. In particular, 5.4% (2 out of 37) of strains were associated with carriage episodes, 32.4% (12 out of 37) with colonization episodes, and 62.2% (23 out of 37) with infections (52.2% pneumonia, 26.1% CVC-related BSIs, and 21.7% BSIs).

### 3.2. Biofilm Formation and Clinical and Epidemiological Features

**Figure 1:**
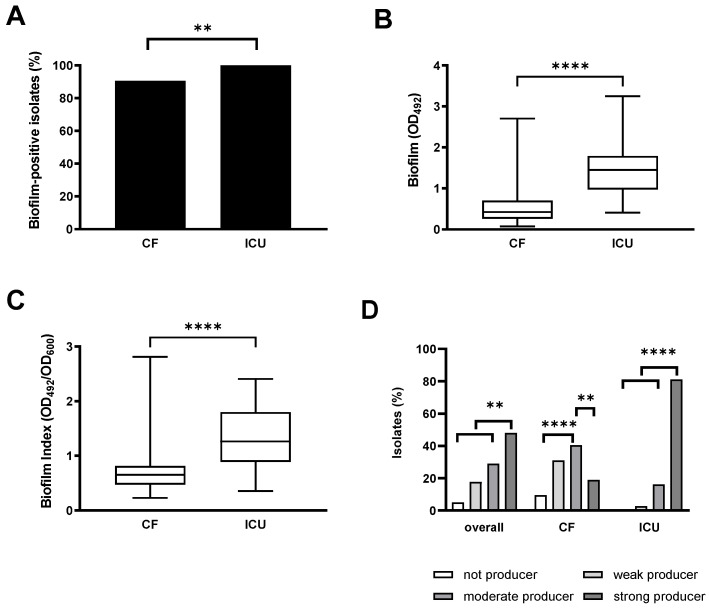
Biofilm formation by *S. maltophilia* strains from CF and ICU patients. (**A**) Prevalence of CF (*n* = 42) and ICU (*n* = 37) strains able to form biofilm; ** *p* < 0.01, Fisher’s exact test. (**B**,**C**) Biofilm biomass amount and Biofilm Index by CF and ICU strains. Results are shown as box-and-whisker plots, where the box represents the interquartile range from the first to the third quartiles, with the median indicated by the line inside the box, and the whiskers extend to the minimum and maximum values. **** *p* < 0.0001, unpaired *t*-test. (**D**) Prevalence of biofilm-based groups considering strains as a whole, CF strains, and ICU strains; from left to right, the groups, according to Stepanovic et al. [27], are: not-producer, weak-producer, moderate-producer, and strong-producer. ** *p* < 0.01, and **** *p* < 0.0001, Fisher’s exact test.

The ability to form biofilm was more prevalent in ICU strains than CF ones (100% vs. 90.5%, respectively; *p* < 0.01) (Figure 1A). ICU strains formed a mean biofilm biomass significantly higher compared to that formed by CF ones (OD_492_, mean ± SD: 1.454 ± 0.679 vs. 0.550 ± 0.449, respectively; *p* < 0.0001) (Figure 1B). The same trend was observed after normalization of biofilm biomass amount on the planktonic growth, as assessed by the Biofilm Index (mean ± SD: 1.335 ± 0.587 vs. 0.720 ± 0.419, respectively, for ICU and CF strains; *p* < 0.0001) (Figure 1C).

Stratifying biofilm biomass amount according to the criteria proposed by Stepanovic et al. [27], the “strong-producer” class was significantly higher than other groups overall (*p* < 0.01) and ICU strains (*p* < 0.0001) (Figure 1D). In contrast, among CF strains, the “moderate-producer” group was more prevalent than the “non-producer” (*p* < 0.0001) and “strong-producer” (*p* < 0.01) groups, although comparable to the “weak-producer” group (Figure 1D).

**Figure 2:**
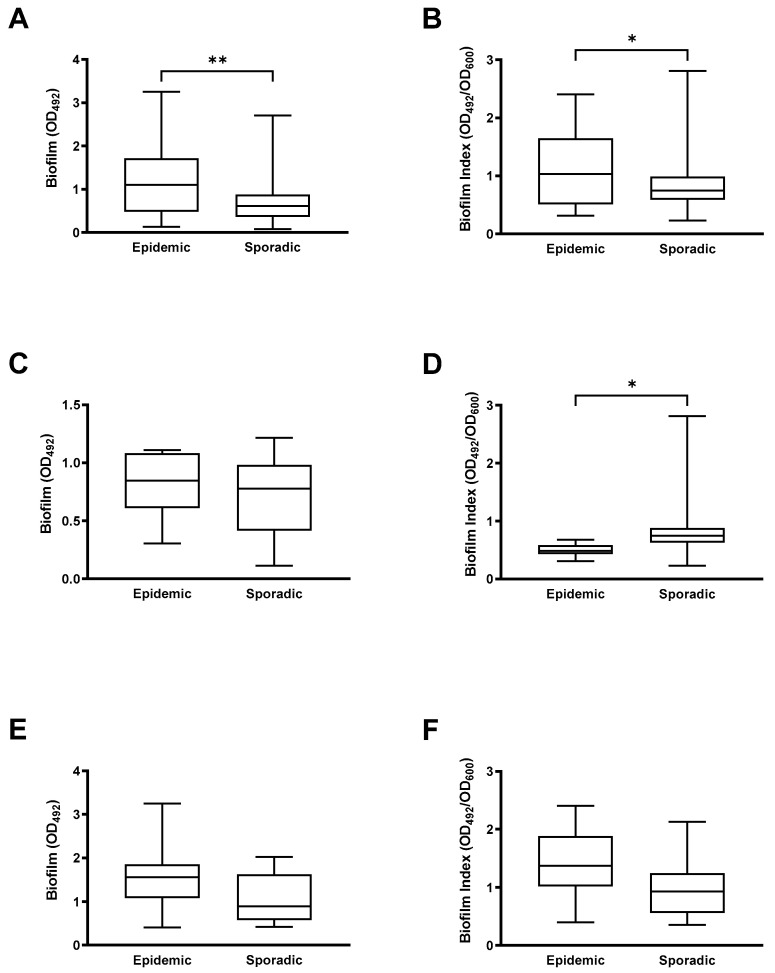
Biofilm formation by epidemic and sporadic *S. maltophilia* strains. Biofilm biomass amount and Biofilm Index for (**A**,**B**) strains as a whole, (**C**,**D**) CF strains, and (**E**,**F**) ICU strains. Results are shown as box-and-whisker plots, where the box represents the interquartile range from the first to the third quartiles, with the median indicated by the line inside the box, and the whiskers extend to the minimum and maximum values. * *p* < 0.05, and ** *p* < 0.01, unpaired *t*-test.

Considering the strains as a whole (*n* = 79), the mean amount of biofilm biomass formed was shown to be significantly higher in epidemic than in sporadic ones (OD_492_, mean ± SD: 1.186 ± 0.777 vs. 0.720 ± 0.569, respectively; *p* < 0.01) (Figure 2A). No significant differences were observed when biofilm formation by epidemic and sporadic strains was stratified by CF or ICU setting (Figure 2C,E). Biofilm Index values confirmed the same trend when strains were considered overall (mean ± SD: 1.127 ± 0.640 vs. 0.865 ± 0.493 for epidemic and sporadic strains, respectively; *p* < 0.05) (Figure 2B) or in ICU settings (mean ± SD: 1.430 ± 0.564 vs. 0.989 ± 0.572, respectively; *p* > 0.05) (Figure 2F). Conversely, among CF strains, the Biofilm Index was significantly higher for sporadic than for epidemic strains (mean ± SD: 0.499 ± 0.095 vs. 0.830 ± 0.473 for epidemic and sporadic strains, respectively; *p* < 0.05) (Figure 2D).

**Figure 3:**
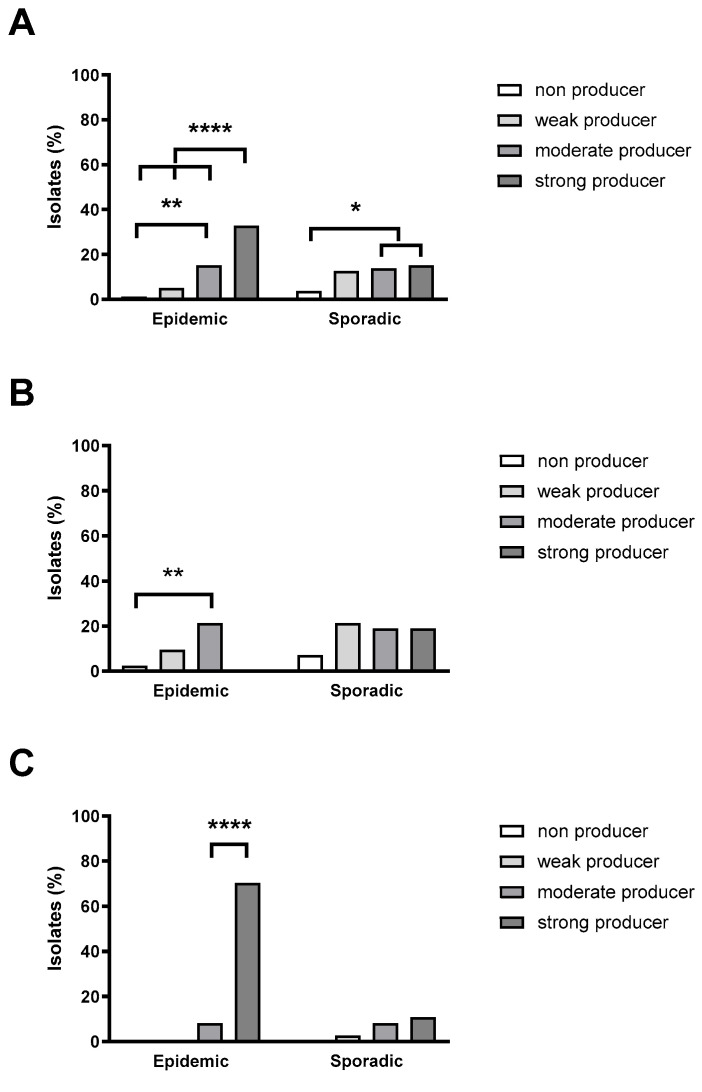
Prevalence of biofilm-based groups among epidemic and sporadic *S. maltophilia* strains. Prevalence of biofilm classes considering (**A**) strains as a whole, (**B**) CF strains, and (**C**) ICU strains. From left to right, the groups, according to Stepanovic et al. [27], are: not-producer, weak-producer, moderate-producer, and strong-producer. * *p* < 0.05, ** *p* < 0.01, and **** *p* < 0.0001, Fisher’s exact test.

Overall, “strong-producer” was the most prevalent biofilm class, although to a greater extent among epidemic (32.9%; *p* < 0.0001 vs. other classes) than among sporadic (15.2%; *p* < 0.05 vs. “non-producer”) strains (Figure 3A). Stratification in clinical settings revealed that “strong-producer” strains were the most prevalent among ICU epidemic strains (70.3%; *p* < 0.0001 vs. other classes) (Figure 3C). Conversely, no epidemic CF strains were classified as a “strong producer” (Figure 3B). No statistically significant differences were observed among sporadic strains across clinical settings (Figure 3B,C).

**Figure 4:**
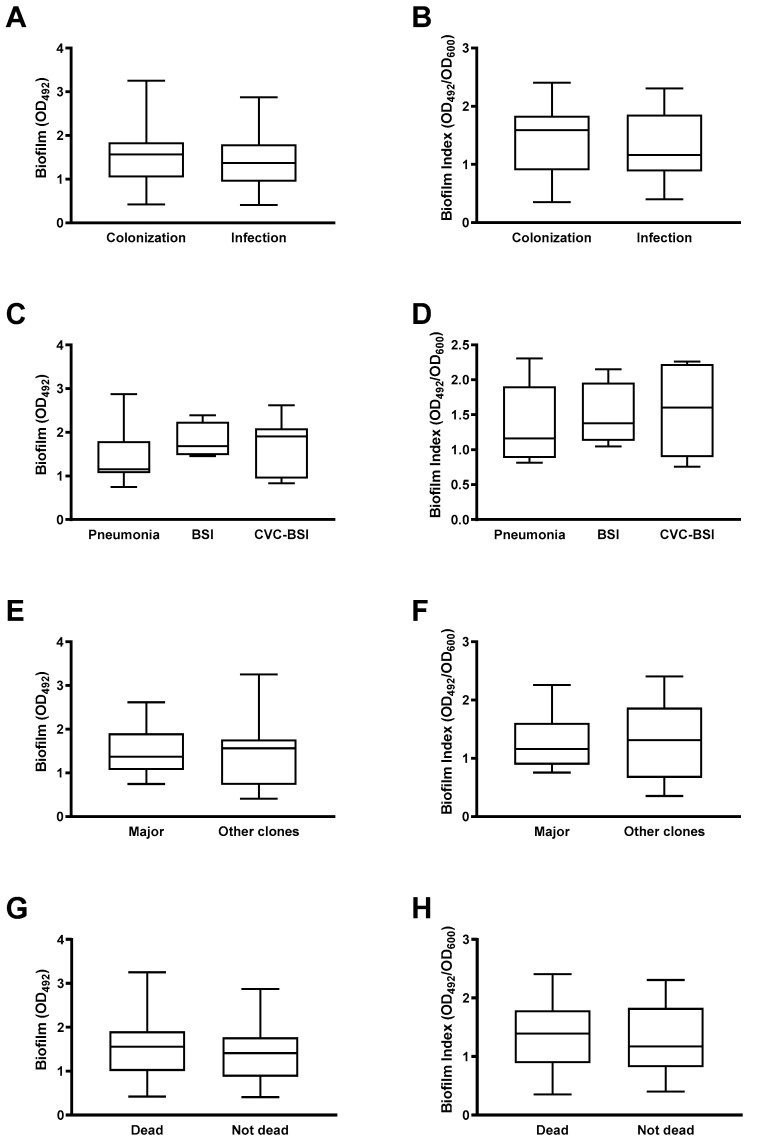
Biofilm and clinical and epidemiological features in *S. maltophilia* strains from ICU patients. Biofilm biomass amount and Biofilm Index for strains (**A**,**B**) causing colonization or infection; (**C**,**D**) causing pneumonia, bloodstream infections (BSI), or CVC-BSIs; (**E**,**F**) belonging to the major clone A or other (minor) clones; and (**G**,**H**) isolated from patients, dead or not dead. Results are shown as box-and-whisker plots, where the box represents the interquartile range from the first to the third quartiles, with the median indicated by the line inside the box, and the whiskers extend to the minimum and maximum values. Unpaired *t*-test showed no statistically significant differences.

Regarding ICU-associated *S. maltophilia* strains, no significant differences were observed in the mean biofilm amount formed by colonization-associated and infection-associated strains (OD_492_; mean ± SD: 1.565 ± 0.793 vs. 1.413 ± 0.644, respectively; *p* > 0.05) (Figure 4A). Similarly, no differences were observed among pneumonia, BSI-related, and CVC-BSI infection (OD_492_, mean ± SD: 1.499 ± 0.714 vs. 1.801 ± 0.415 vs. 1.683 ± 0.654, respectively; *p* > 0.05) (Figure 4C), between major clone A strains and those belonging to other clones (OD_492_, mean ± SD: 1.481 ± 0.569 vs. 1.436 ± 0.758, respectively; *p* > 0.05) (Figure 4E), and about fatal outcome during ICU stay (OD_492_, mean ± SD: 1.527 ± 0.723 vs. 1.415 ± 0.667, for dead and alive patients, respectively; *p* > 0.05) (Figure 4G). These trends were confirmed after calculating the Biofilm Index (Figure 4B,D,F,H).

**Figure 5:**
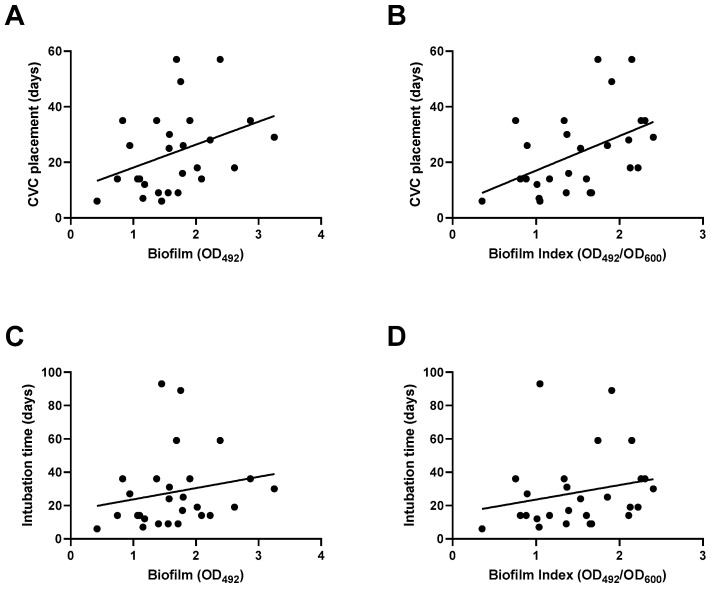
Biofilm formation and intubation/CVC duration. Linear regression between biofilm formation or Biofilm Index and (**A**,**B**) CVC placement duration, or (**C**,**D**) intubation time in ICU strains. A significant relationship was observed between the Biofilm Index and CVC placement duration (Pearson r: 0.470; *p* < 0.05).

The mean biofilm amount was not significantly associated with duration of CVC (Figure 5A) or intubation (Figure 5B). The Biofilm Index was statistically correlated with CVC duration only (Pearson r: 0.470; *p* < 0.05) (Figure 5B).

### 3.3. Biofilm and Motility

**Figure 6:**
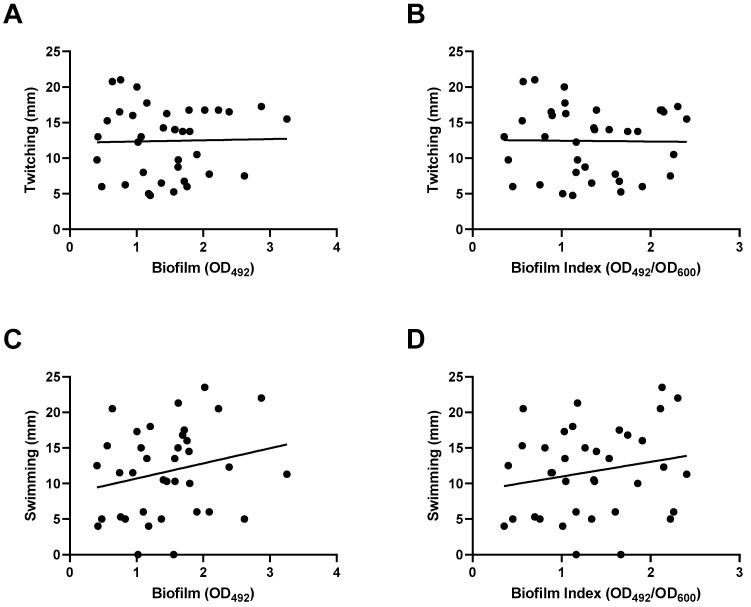
Biofilm and motility in *S. maltophilia* strains from ICU patients. Linear regression between biofilm formation or Biofilm Index and (**A**,**B**) twitching motility, or (**C**,**D**) swimming motility of 37 ICU strains. Motility assays were performed as described by Rashid et al. [28]. Simple linear regression analysis showed no significant relationship.

Swimming and twitching motility assays were performed only on ICU-associated isolates, as this subset exhibited the strongest biofilm phenotype and was the primary population of interest for exploring phenotypic traits potentially associated with biofilm-mediated persistence in the critical care setting. Results are shown in Figure 6.

Both motility types were highly conserved among 37 ICU strains, albeit to varying degrees. Swimming was observed in all but the M9 strain (36 out of 37, 97.3%), ranging from 0 to 23.5 mm (mean ± SD: 11.6 ± 6.2 mm). All the strains showed twitching motility, ranging from 4.8 to 21 mm (mean ± SD: 12.5 ± 4.8 mm). Simple linear regression analysis indicated no relationship between biofilm amount or Biofilm Index and swimming or twitching motilities (Figure 6B,D).

### 3.4. Clonality Assessment and Genetic Diversity

**Figure 7:**
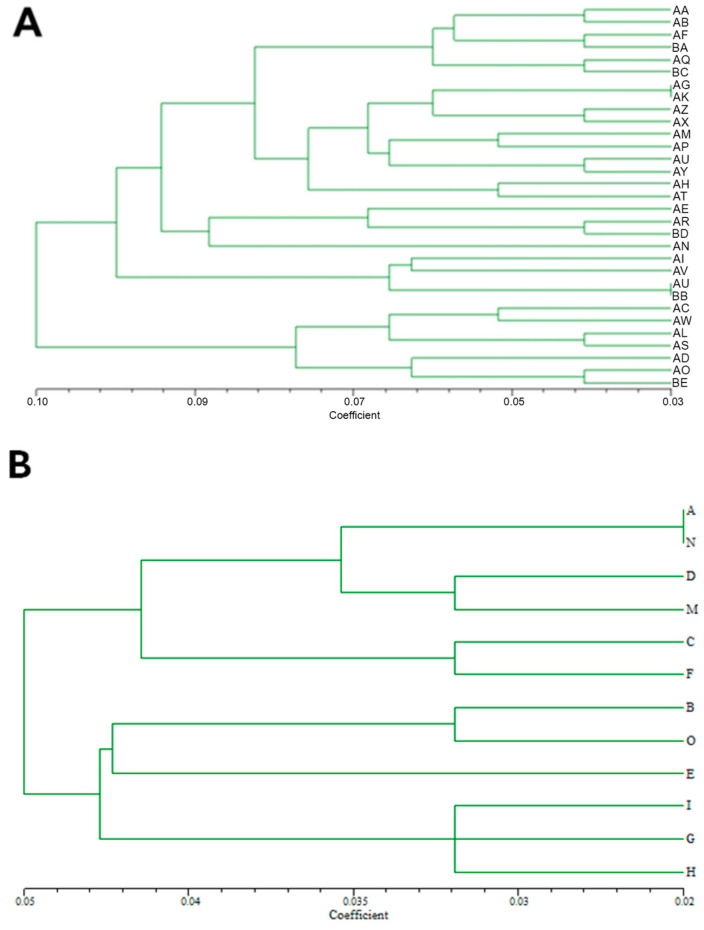
Hierarchical clustering of *S. maltophilia* strains. Dendrograms were generated based on the calculated similarity/distance coefficient reported on the *x*-axis. Terminal branches correspond to individual profiles/isolates. The clustering of profiles for (**A**) CF and (**B**) ICU *S. maltophilia* strains is shown. Shorter branch lengths indicate higher similarity among profiles, while greater separation along the coefficient axis indicates increasing dissimilarity. PFGE types are indicated on the right side.

Categories of genetic and epidemiological relatedness observed in ICU and CF strains at PFGE analysis are shown in Figure 7. High genetic diversity was observed among *S. maltophilia* strains, particularly among CF strains. In fact, the genetic diversity index of *S. maltophilia* isolated in CF patients (73.8%, 31 different PFGE profiles among 42 strains tested) was significantly higher (*p* < 0.0001) than that observed for *S. maltophilia* isolated in ICU patients (35.1%, 13 different PFGE profiles among 37 strains tested).

Among 42 CF strains tested, 3 identical PFGE types were shared by 2 or more isolates, likely associated with cross-transmission in 14 episodes (33.3%), and a major clone was responsible for the epidemic spread of *S. maltophilia* in 10 of 42 (23.8%) CF strains. Among 37 ICU strains tested, 4 identical PFGE types were associated with a total of 23 cross-transmission episodes (62.2%), with a major clone responsible for the epidemic spread of *S. maltophilia* in 15 out of 37 (40.5%) of ICU patients. The impact of cross-transmission due to *S. maltophilia* acquisition was higher across all ICU episodes than across all CF infections, and this difference was statistically significant (*p* < 0.001). No PFGE types were shared between CF and ICU strains.

## 4. Discussion

The present study shows that *S. maltophilia* behaves differently in ICU and CF settings. Compared with CF isolates, ICU strains were less genetically diverse, were more frequently involved in clonal clustering/cross-transmission episodes, and exhibited a stronger biofilm-forming phenotype. Importantly, this difference was confirmed not only by the absolute crystal violet-stained biomass, but also after normalization for planktonic growth using the Biofilm Index. This normalization step reduces the likelihood that the ICU-CF difference reflects unequal planktonic growth. Conversely, CF isolates showed greater heterogeneity and lower average biofilm formation, although most retained the ability to produce biofilm. These observations are consistent with previous reports describing *S. maltophilia* as an opportunistic pathogen with distinct epidemiological patterns in hospital and chronic airway settings [1,2]. In the ICU, circulation may involve a limited number of successful hospital-associated genetic lineages. In contrast, in CF, isolation may more often reflect repeated acquisition and/or diversification within chronically infected airways under prolonged antibiotic and host-selective pressures [7,15]. The possible contribution of environmental or water-associated sources to ICU persistence also remains biologically plausible, as suggested by previous outbreak investigations [20,29,35].

The ICU subset showed the clearest convergence between clonal clustering and enhanced biofilm formation, although this association should be interpreted cautiously. The predominance of a few PFGE types, the universal ability of ICU isolates to form biofilm, and the enrichment of the “strong-producer” biofilm phenotype are consistent with the hypothesis that biofilm formation may contribute to the persistence of selected *S. maltophilia* lineages in the critical care setting. Biofilm formation is a well-recognized mechanism of microbial survival on abiotic surfaces and medical devices [11]. The fact that ICU isolates retained significantly higher Biofilm Index values than CF isolates suggests that these strains may be more efficient at producing surface-associated biomass than their planktonic counterparts. In critically ill patients exposed to invasive devices, mechanical ventilation, broad-spectrum antibiotics, and prolonged hospitalization, such a phenotype may favor survival on abiotic surfaces, patient-associated devices, and hospital surfaces [12]. However, because environmental surveillance was not performed, our data do not allow us to identify specific reservoirs in water sources, ventilator equipment, intravascular devices, or other ICU-associated niches, nor do they demonstrate direct transmission from environmental sources to patients. Therefore, the association observed between clonal clustering and enhanced biofilm formation should be regarded as biologically plausible rather than as definitive evidence of environmental persistence or nosocomial dissemination. This interpretation is consistent with previous reports implicating water-associated reservoirs in *S. maltophilia* persistence [20], as well as studies highlighting the role of humid hospital niches, ventilator equipment, and intravascular devices in transmission or device-associated infections [36,37,38].

The comparison between epidemic and sporadic isolates further supports a setting-dependent interpretation. When isolates were analyzed as a whole, epidemic strains formed significantly more biofilm than sporadic strains, and this difference persisted after normalization with the Biofilm Index. However, when the analysis was stratified by clinical setting, differences between epidemic and sporadic isolates were no longer statistically significant in either the CF or ICU groups. This suggests that the overall association between epidemic status and biofilm formation is at least partly driven by the distribution of ICU isolates within clonal clusters, rather than by a universal relationship between epidemic behavior and biofilm biomass across all clinical contexts. Therefore, biofilm formation may contribute to the success of specific hospital-associated lineages, but it should not be considered the only determinant of clonal spread. Other factors, including antimicrobial exposure, host vulnerability, device use, environmental persistence, infection-control practices, and strain-specific genetic traits, are likely to interact with biofilm formation in shaping the epidemiology of *S. maltophilia*.

The CF subset followed a different, more complex pattern. Biofilm formation remained common among CF-associated isolates, but these strains showed lower biomass and Biofilm Index values than ICU isolates. Moreover, epidemic CF isolates were not enriched among strong biofilm producers, suggesting that persistence in CF is not explained solely by biofilm biomass on polystyrene. The static crystal violet assay measures total surface-associated biomass under simplified in vitro conditions. Still, it does not capture the full complexity of the CF airway, including mucus composition, host-derived substrates, inflammation, polymicrobial interactions, antimicrobial exposure, and longitudinal within-host adaptation. Previous studies have shown that *S. maltophilia* may persist in CF airways and form biofilm on abiotic surfaces [14]. Biofilm formation has also been demonstrated on CF-derived epithelial cells, supporting a possible role in airway adaptation [15,16]. At the same time, genomic and clinical studies suggest that *S. maltophilia* in CF may often arise from independent acquisition or from indirect, shared sources rather than direct patient-to-patient transmission [35]. Chronic infection has been associated in several cohorts with lung function decline and increased hospitalization [7,8]. However, the overall clinical role of this organism remains debated and may depend on host, microbial, and longitudinal factors [39,40].

Among ICU isolates, biofilm formation was not significantly associated with colonization or infection, with specific infection type, with major clone, or with fatal outcome. These findings were confirmed by analysis of biofilm using the Biofilm Index, which indicated that growth-normalized biofilm-forming efficiency did not discriminate among most clinical subgroups. This lack of association should be interpreted cautiously. First, the sample size was limited once ICU isolates were stratified by clinical presentation, infection type, clonal profile, or outcome. Second, biofilm formation was highly prevalent among ICU isolates, which may reduce its ability to distinguish clinical severity once a high-biofilm phenotype is already common. Third, host factors, illness severity, concomitant pathogens, antimicrobial exposure, and device management likely modulate whether colonization progresses to infection or whether infection affects outcome. Accordingly, biofilm formation may be better viewed as a facilitator of initial colonization or persistence, and of device-related or invasive infection, rather than as a stand-alone predictor of infection severity and outcome [36,37,38].

Interestingly, the Biofilm Index, but not absolute biofilm biomass, showed a significant positive correlation with CVC duration. This observation suggests that growth-adjusted biofilm formation may capture a device-related signal not evident from crude OD_492_ values alone. One possible interpretation is that longer CVC placement may create selective conditions favoring strains with a greater ability to form biofilm than to grow planktonically, or that such strains are more likely to persist in patients requiring prolonged intravascular access. However, this finding remains exploratory. The correlation does not prove causality, and significant differences between CVC-related bloodstream infection isolates and other infection groups did not accompany it. Therefore, the association between Biofilm Index and CVC duration should be interpreted as a hypothesis-generating observation that supports further investigation in larger, prospective studies focused on device-associated *S. maltophilia* colonization and infection.

Given the potential contribution of bacterial motility to early surface colonization and biofilm development, swimming and twitching motility were evaluated in ICU-associated isolates. Neither swimming nor twitching motility was significantly associated with absolute biofilm biomass or with the Biofilm Index. These results suggest that the enhanced biofilm phenotype of ICU-associated isolates is not explained solely by motility or by growth-normalized surface accumulation. Biofilm formation in *S. maltophilia* is increasingly recognized as a multifactorial process involving surface appendages, adhesins, and extracellular matrix composition [37]. LPS/EPS-related pathways and diffusible signal factor-mediated regulation may also contribute to biofilm development and adaptation [41,42]. Thus, although flagella and type IV pili may contribute to early surface colonization or biofilm architecture, they did not predict total biofilm biomass or Biofilm Index under the in vitro conditions tested in the present study.

This study has some limitations. First, the study was retrospective and included a relatively small number of isolates, particularly after stratification by clinical setting, infection type, clonal profile, or outcome. Second, clonality was assessed by PFGE rather than more robust and accurate genotypic methods, such as whole-genome sequencing (WGS). Although PFGE remains useful for investigating clonal spread over short time periods, its discriminatory power is lower than that of WGS, and it may overestimate epidemiologically relatedness among isolates. MLST would have provided a more reproducible population structure, but it may not have sufficient discriminatory power to infer short-term epidemiological links. Third, environmental sampling was not performed, preventing direct demonstration of reservoirs in water sources, ventilator equipment, catheters, or other ICU-associated niches. Fourth, biofilm was evaluated using a static crystal violet assay on polystyrene. This useful but simplified model does not fully reproduce the biological complexity of respiratory epithelium, bloodstream infection, or indwelling devices. Although the Biofilm Index helped control for growth-related bias, it remains based on endpoint planktonic growth, and it does not capture biofilm viability, matrix composition, architecture, or behavior under flow conditions. Future studies using environmental surveillance, longitudinal patient sampling, WGS, device-associated models, flow-cell systems, cell culture, or organ-on-chip approaches will be needed to clarify the mechanisms linking biofilm formation, persistence, and transmission in *S. maltophilia* populations.

## 5. Conclusions

The findings from the present study indicate that *S. maltophilia* isolates from ICU and CF settings differ in biofilm-forming capacity, clonal structure, and epidemiological behavior. ICU-associated isolates showed reduced genetic diversity, more frequent clonal clustering/cross-transmission episodes, and higher biofilm formation than CF isolates. Importantly, this difference was confirmed after normalization for planktonic growth using the Biofilm Index, supporting the view that ICU isolates display a stronger growth-adjusted biofilm-forming phenotype.

These findings suggest that *S. maltophilia* should not be regarded solely as an incidental colonizer in critically ill patients, but as an opportunistic pathogen with the potential to persist and disseminate within high-risk hospital settings. The enrichment of strong biofilm-forming lineages among ICU isolates is compatible with a possible role of biofilm in environmental persistence, device-associated colonization/infection, and clonal spread. However, because environmental surveillance was not performed, the present data do not establish a direct link between clinical isolates, environmental reservoirs, and transmission routes.

From a clinical and infection-control perspective, these results support the importance of microbiological surveillance, early recognition of clonal clusters, careful management of indwelling devices, and attention to water-associated hospital niches in ICUs where *S. maltophilia* is repeatedly isolated. At the same time, the distinct behavior of CF isolates highlights that conclusions drawn from ICU populations cannot be automatically generalized to other clinical settings. Future studies integrating environmental surveillance, longitudinal patient sampling, device-associated biofilm models, and high-resolution genomic typing will be needed to clarify the mechanisms linking biofilm formation, persistence, and transmission in *S. maltophilia* populations.

## Figures and Tables

**Table 1:** Microbiological and clinical features of the 79 *S. maltophilia* strains.

Strain ID	Ward	Sample ^a^	Infection Type ^b^	PFGE ^c^ Type	Major Clone	Mean Biofilm (OD_492_)	Biofilm Class ^d^	Intubation Duration (Days) ^e^	CVC Duration (Days) ^f^	Death in the ICU ^g^
SM103	CF	sputum	pneumonia	AC	no	0.483	M	none	none	
SM104	CF	sputum	pneumonia	AD	no	0.463	M	none	none	
SM105	CF	sputum	pneumonia	AE	no	0.669	M	none	none	
SM106	CF	sputum	pneumonia	AA	no	0.572	M	none	none	
SM107	CF	sputum	pneumonia	AF	no	0.883	S	none	none	
SM108	CF	sputum	pneumonia	AG	no	0.286	W	none	none	
SM109	CF	sputum	pneumonia	AH	no	0.904	S	none	none	
SM110	CF	sputum	pneumonia	AA	no	0.613	M	none	none	
SM111	CF	sputum	pneumonia	AB	no	0.259	W	none	none	
SM112	CF	sputum	pneumonia	AB	no	0.166	N	none	none	
SM113	CF	sputum	pneumonia	AI	no	0.111	N	none	none	
SM114	CF	sputum	pneumonia	AL	no	0.162	N	none	none	
SM115	CF	sputum	pneumonia	AM	no	0.390	W	none	none	
SM116	CF	sputum	pneumonia	AN	no	0.398	W	none	none	
SM117	CF	sputum	pneumonia	AO	no	0.148	N	none	none	
SM118	CF	sputum	pneumonia	AP	no	0.948	S	none	none	
SM119	CF	sputum	pneumonia	AX	no	0.733	M	none	none	
SM120	CF	sputum	pneumonia	AQ	no	0.426	W	none	none	
SM122	CF	sputum	pneumonia	AR	no	1.589	S	none	none	
SM123	CF	sputum	pneumonia	AS	no	0.913	S	none	none	
SM124	CF	sputum	pneumonia	AT	no	0.276	W	none	none	
SM130	CF	sputum	pneumonia	AY	no	0.766	M	none	none	
SM134	CF	sputum	pneumonia	AA	no	0.694	M	none	none	
SM135	CF	sputum	pneumonia	AA	no	0.692	M	none	none	
SM136	CF	sputum	pneumonia	AA	no	0.645	M	none	none	
SM137	CF	sputum	pneumonia	AU	no	0.910	S	none	none	
SM138	CF	sputum	pneumonia	AV	no	0.434	M	none	none	
SM139	CF	sputum	pneumonia	AZ	no	0.922	S	none	none	
SM140	CF	sputum	pneumonia	AJ	no	0.399	W	none	none	
SM142	CF	sputum	pneumonia	AK	no	2.742	S	none	none	
SM144	CF	sputum	pneumonia	BA	no	0.630	M	none	none	
SM150	CF	sputum	pneumonia	BB	no	0.793	M	none	none	
SM156	CF	sputum	pneumonia	BC	no	0.243	W	none	none	
SM157	CF	sputum	pneumonia	BD	no	0.253	W	none	none	
SM159	CF	sputum	pneumonia	BE	no	0.395	W	none	none	
SM189	CF	sputum	pneumonia	AW	no	0.430	M	none	none	
SM190	CF	sputum	pneumonia	AA	no	0.415	M	none	none	
SM191	CF	sputum	pneumonia	AW	no	0.459	M	none	none	
SM192	CF	sputum	pneumonia	AA	no	0.512	M	none	none	
SM193	CF	sputum	pneumonia	AA	no	0.334	W	none	none	
SM194	CF	sputum	pneumonia	AA	no	0.252	W	none	none	
SM195	CF	sputum	pneumonia	AA	no	0.301	W	none	none	
405	ICU	blood	BSI	C	no	2.059	S	19	19	yes
1100 (2)	ICU	BAL	AC	D	no	1.440	S	9	9	yes
1196 (3)	ICU	urinary catheter	urinary catheter colonization	D	no	1.752	S	9	9	yes
72 (2)	ICU	lower limb swab	skin colonization	O	no	0.459	W	6	6	yes
591 (2)	ICU	BAL	airway colonization	D	no	1.727	S	59	39	not
165 (2)	ICU	BAL	airway colonization	B	no	2.264	S	28	28	not
1262 (2)	ICU	BAL	airway colonization	D	no	1.610	S	23	25	not
589	ICU	BAL	airway colonization	B	no	3.291	S	29	29	not
1162	ICU	BAL	airway colonization	D	no	1.594	S	9	9	yes
419 (1)	ICU	BAL	pneumonia	B	no	2.907	S	35	35	not
1091 (2)	ICU	BAL	pneumonia	D	no	1.833	S	25	26	not
1257 (1)	ICU	BAL	pneumonia	D	no	1.794	S	89	88	yes
494	ICU	blood	CVC-related bacteremia	A	yes	2.127	S	14	25	yes
501	ICU	blood	CVC-related bacteremia	A	yes	1.941	S	36	35	not
515 (2)	ICU	venous catheter	CVC-related bacteremia	A	yes	0.869	S	36	35	not
407	ICU	blood	CVC-related bacteremia	A	yes	0.981	S	27	26	yes
481 (2)	ICU	blood	CVC-related bacteremia	A	yes	2.655	S	19	19	not
426 (2)	ICU	blood	CVC-related bacteremia	A	yes	1.411	S	36	35	not
409	ICU	blood	BSI	A	yes	1.489	S	3	6	not
499	ICU	blood	BSI	A	yes	1.615	S	31	31	not
585	ICU	blood	BSI	A	yes	2.426	S	59	39	not
482 (2)	ICU	blood	BSI	A	yes	1.823	S	17	16	yes
221	ICU	BAL	airways colonization	A	yes	1.217	S	12	12	not
434 (2)	ICU	BAL	pneumonia	A	yes	0.784	M	14	25	yes
656 (3)	ICU	BAL	pneumonia	A	yes	1.193	S	7	7	not
435 (2)	ICU	BAL	pneumonia	A	yes	1.139	S	14	25	yes
445	ICU	pleural fluid	pneumonia	A	yes	1.104	S	14	25	yes
72A-288/15197	ICU	wound swab	skin colonization	F	no	1.244	S	NA	NA	NA
9M	ICU	BAL	pneumonia	H	no	1.058	S	NA	NA	NA
0606/1036	ICU	BAL	pneumonia	G	no	1.658	S	NA	NA	NA
73A-1010/14899	ICU	BAL	pneumonia	E	no	0.515	M	NA	NA	NA
14117/666	ICU	stoma swab	skin colonization	M	no	0.597	M	NA	NA	NA
21/636	ICU	BAL	pneumonia	L	no	0.675	M	NA	NA	NA
01/01597	ICU	pharynx swab	airways colonization	I	no	1.664	S	NA	NA	NA
68A-286/15107	ICU	wound swab	skin colonization	F	no	1.043	S	NA	NA	NA
43A-13707	ICU	BAL	pneumonia	E	no	0.445	M	NA	NA	NA
29A-13232	ICU	nasal swab	skin colonization	N	no	0.797	M	NA	NA	NA

^a^ BAL, bronchoalveolar lavage. ^b^ BSI, bloodstream infection; CVC, central venous catheter. ^c^ PFGE, pulsed-field gel electrophoresis. ^d^ N, non-producer (OD_492_ < 0.200); W, weak-producer (0.200 ≤ OD_492_ < 0.400); M, moderate-producer (0.400 ≤ OD_492_ < 0.800); S, strong-producer (OD_492_ ≥ 0.800). ^e,f,g^ NA, not available.

## Data Availability

The raw data supporting the conclusions of this article will be made available by the authors on request.

## Abbreviations

The following abbreviations are used in this manuscript:

ICU	Intensive Care Unit
CF	Cystic Fibrosis
CDC	Centers for Disease Control
BSIs	Bloodstream Infections
CVC	Central Venous Catheter
SSIs	Surgical Site Infections
MHA	Mueller-Hinton Agar
TSB	Trypticase Soy Broth
PBS	Phosphate-Buffered Saline
OD_492_	Optical Density at 492 nm wavelength
OD_600_	Optical Density at 600 nm wavelength
PFGE	Pulsed-Field Gel Electrophoresis
LPS	Lipopolysaccharide
EPS	Extracellular Polymeric Substance
WGS	Whole-Genome Sequencing
MLST	Multi Locus Sequence Typing

## References

[1] Barchitta M., Cipresso R., Giaquinta L., Romeo M.A., Denaro C., Pennisi C., Agodi A. (2009). Acquisition and spread of *Acinetobacter baumannii* and *Stenotrophomonas maltophilia* in intensive care patients. Int. J. Hyg. Environ. Health.

[2] Saugel B., Eschermann K., Hoffmann R. (2012). *Stenotrophomonas maltophilia* in the respiratory tract of medical intensive care unit patients. Eur. J. Clin. Microbiol. Infect. Dis..

[3] Zuschneid I., Schwab F., Geffers C., Behnke M., Rüden H., Gastmeier P. (2007). Trends in ventilator-associated pneumonia rates within the German nosocomial infection surveillance system (KISS). Infect. Control Hosp. Epidemiol..

[4] Agodi A., Auxilia F., Barchitta M., Brusaferro S., D’Alessandro D., Montagna M.T., Orsi G.B., Pasquarella C., Torregrossa V., Suetens C. (2010). Building a benchmark through active surveillance of ICU-acquired infections: The Italian network SPIN-UTI. J. Hosp. Infect..

[5] European Centre for Disease Prevention and Control (2026). Healthcare-associated infections acquired in intensive care units. ECDC Annual Epidemiological Report for 2022.

[6] Davies J.C., Rubin B.K. (2007). Emerging and unusual gram-negative infections in cystic fibrosis. Semin. Respir. Crit. Care Med..

[7] Ciofu O., Hansen C.R., Høiby N. (2013). Respiratory bacterial infections in cystic fibrosis. Curr. Opin. Pulm. Med..

[8] Terlizzi V., Tomaselli M., Giacomini G., Dalpiaz I., Chiappini E. (2023). *Stenotrophomonas maltophilia* in people with Cystic Fibrosis: A systematic review of prevalence, risk factors and management. Eur. J. Clin. Microbiol. Infect. Dis..

[9] Sánchez M.B. (2015). Antibiotic resistance in the opportunistic pathogen *Stenotrophomonas maltophilia*. Front. Microbiol..

[10] Gil-Gil T., Martínez J.L., Blanco P. (2020). Mechanisms of antimicrobial resistance in *Stenotrophomonas maltophilia*: A review of current knowledge. Expert Rev. Anti-Infect. Ther..

[11] de la Fuente-Núñez C., Reffuveille F., Fernández L., Hancock R.E. (2013). Bacterial biofilm development as a multicellular adaptation: Antibiotic resistance and new therapeutic strategies. Curr. Opin. Microbiol..

[12] Van Acker H., Van Dijck P., Coenye T. (2014). Molecular mechanisms of antimicrobial tolerance and resistance in bacterial and fungal biofilms. Trends Microbiol..

[13] Di Bonaventura G., Spedicato I., D’Antonio D., Robuffo I., Piccolomini R. (2004). Biofilm formation by *Stenotrophomonas maltophilia*: Modulation by quinolones, trimethoprim-sulfamethoxazole, and ceftazidime. Antimicrob. Agents Chemother..

[14] Di Bonaventura G., Prosseda G., Del Chierico F., Cannavacciuolo S., Cipriani P., Petrucca A., Superti F., Ammendolia M.G., Concato C., Fiscarelli E. (2007). Molecular characterization of virulence determinants of *Stenotrophomonas maltophilia* strains isolated from patients affected by cystic fibrosis. Int. J. Immunopathol. Pharmacol..

[15] Pompilio A., Pomponio S., Crocetta V., Gherardi G., Verginelli F., Fiscarelli E., Dicuonzo G., Savini V., D’Antonio D., Di Bonaventura G. (2011). Phenotypic and genotypic characterization of *Stenotrophomonas maltophilia* strains from patients with cystic fibrosis: Genome diversity, biofilm formation, and virulence. BMC Microbiol..

[16] Pompilio A., Crocetta V., Confalone P., Nicoletti M., Petrucca A., Guarnieri S., Fiscarelli E., Savini V., Piccolomini R., Di Bonaventura G. (2010). Adhesion to and biofilm formation on IB3-1 bronchial cells by *Stenotrophomonas maltophilia* isolates from cystic fibrosis patients. BMC Microbiol..

[17] Pompilio A., Crocetta V., Ghosh D., Chakrabarti M., Gherardi G., Vitali L.A., Fiscarelli E., Di Bonaventura G. (2016). *Stenotrophomonas maltophilia* Phenotypic and Genotypic Diversity during a 10-year Colonization in the Lungs of a Cystic Fibrosis Patient. Front. Microbiol..

[18] de Oliveira-Garcia D., Dall’Agnol M., Rosales M., Azzuz A.C., Alcantara N., Martinez M.B., Giron J.A. (2003). Fimbriae and adherence of *Stenotrophomonas maltophilia* to epithelial cells and to abiotic surfaces. Cell. Microbiol..

[19] Pompilio A., Piccolomini R., Picciani C., D’Antonio D., Savini V., Di Bonaventura G. (2008). Factors associated with adherence to and biofilm formation on polystyrene by *Stenotrophomonas maltophilia*: The role of cell surface hydrophobicity and motility. FEMS Microbiol. Lett..

[20] Guyot A., Turton J.F., Garner D. (2013). Outbreak of *Stenotrophomonas maltophilia* on an intensive care unit. J. Hosp. Infect..

[21] Verweij P.E., Meis J.F., Christmann V., Van der Bor M., Melchers W.J., Hilderink B.G., Voss A. (1998). Nosocomial outbreak of colonization and infection with *Stenotrophomonas maltophilia* in preterm infants associated with contaminated tap water. Epidemiol. Infect..

[22] Sakhnini E., Weissmann A., Oren I. (2002). Fulminant *Stenotrophomonas maltophilia* soft tissue infection in immunocompromised patients: An outbreak transmitted via tap water. Am. J. Med. Sci..

[23] Garner J.S., Jarvis W.R., Emori T., Horan T.C., Hughes J.M. (1988). CDC definitions for nosocomial infections. Am. J. Infect. Control.

[24] Bertrand X., Thouverez M., Talon D., Boillot A., Capellier G., Floriot C., Hélias J.P. (2001). Endemicity, molecular diversity and colonisation routes of *Pseudomonas aeruginosa* in intensive care units. Intensive Care Med..

[25] Suetens C., Morales I., Savey A., Palomar M., Hiesmayr M., Lepape A., Gastmeier P., Schmit J.C., Valinteliene R., Fabry J. (2007). European surveillance of ICU-acquired infections (HELICS-ICU): Methods and main results. J. Hosp. Infect..

[26] Christensen G.D., Simpson W.A., Younger J.J., Baddour L.M., Barrett F.F., Melton D.M., Beachey E.H. (1985). Adherence of coagulase-negative staphylococci to plastic tissue culture plates: A quantitative model for the adherence of staphylococci to medical devices. J. Clin. Microbiol..

[27] Stepanović S., Vuković D., Hola V., Di Bonaventura G., Djukić S., Cirković I., Ruzicka F. (2007). Quantification of biofilm in microtiter plates: Overview of testing conditions and practical recommendations for assessment of biofilm production by staphylococci. APMIS.

[28] Rashid M.H., Kornberg A. (2000). Inorganic polyphosphate is needed for swimming, swarming, and twitching motilities of *Pseudomonas aeruginosa*. Proc. Natl. Acad. Sci. USA.

[29] Denton M., Todd N.J., Kerr K.G., Hawkey P.M., Littlewood J.M. (1998). Molecular epidemiology of *Stenotrophomonas maltophilia* isolated from clinical specimens from patients with cystic fibrosis and associated environmental samples. J. Clin. Microbiol..

[30] van Belkum A., Tassios P.T., Dijkshoorn L., Haeggman S., Cookson B., Fry N.K., Fussing V., Green J., Feil E., Gerner-Smidt P. (2007). Guidelines for the validation and application of typing methods for use in bacterial epidemiology. Clin. Microbiol. Infect..

[31] Nei M., Lei W.H. (1979). Mathematical model for studying genetic variation in terms of restriction endonucleases. Proc. Natl. Acad. Sci. USA.

[32] Gastmeier P. (2007). Evidence-based infection control in the ICU (except catheters). Curr. Opin. Crit. Care.

[33] Weist K., Pollege K., Schulz I., Rüden H., Gastmeier P. (2002). How many nosocomial infections are associated with cross-transmission? A prospective cohort study in a surgical intensive care unit. Infect. Control Hosp. Epidemiol..

[34] Halwani M., Solaymani-Dodaran M., Grundmann H., Coupland C., Slack R. (2006). Cross-transmission of nosocomial pathogens in an adult intensive care unit: Incidence and risk factors. J. Hosp. Infect..

[35] Izydorczyk C., Waddell B.J., Thornton C.S., Conly J.M., Rabin H.R., Somayaji R., Surette M.G., Church D.L., Parkins M.D. (2023). *Stenotrophomonas maltophilia* natural history and evolution in the airways of adults with cystic fibrosis. Front. Microbiol..

[36] Pompilio A., Ranalli M., Piccirilli A., Perilli M., Vukovic D., Savic B., Krutova M., Drevinek P., Jonas D., Fiscarelli E.V. (2020). Biofilm Formation among *Stenotrophomonas maltophilia* Isolates Has Clinical Relevance: The ANSELM Prospective Multicenter Study. Microorganisms.

[37] Brooke J.S. (2021). Advances in the Microbiology of *Stenotrophomonas maltophilia*. Clin. Microbiol. Rev..

[38] Carbonell N., Oltra M.R., Clari M.Á. (2024). *Stenotrophomonas maltophilia*: The Landscape in Critically Ill Patients and Optimising Management Approaches. Antibiotics.

[39] Waters V., Atenafu E.G., Lu A., Yau Y., Tullis E., Ratjen F. (2013). Chronic *Stenotrophomonas maltophilia* infection and mortality or lung transplantation in cystic fibrosis patients. J. Cyst. Fibros..

[40] Barsky E.E., Williams K.A., Priebe G.P., Sawicki G.S. (2017). Incident *Stenotrophomonas maltophilia* infection and lung function decline in cystic fibrosis. Pediatr. Pulmonol..

[41] García G., Girón J.A., Yañez J.A., Cedillo M.L. (2023). *Stenotrophomonas maltophilia* and its ability to form biofilms. Microbiol. Res..

[42] Bhaumik R., Aungkur N.Z., Anderson G.G. (2024). A guide to *Stenotrophomonas maltophilia* virulence capabilities, as we currently understand them. Front. Cell. Infect. Microbiol..

